# Revisiting Contrail Ice Formation: Impact of Primary Soot Particle Sizes and Contribution of Volatile Particles

**DOI:** 10.1021/acs.est.4c04340

**Published:** 2024-09-26

**Authors:** Fangqun Yu, Bernd Kärcher, Bruce E. Anderson

**Affiliations:** † Atmospheric Sciences Research Center, University at Albany, Albany, New York 12226, United States; ‡ Institut für Physik der Atmosphäre, Deutsches Zentrum für Luft- und Raumfahrt, Oberpfaffenhofen, 82234 Wessling, Germany; § Science Directorate, NASA Langley Research Center, Hampton, Virginia 23666, United States

**Keywords:** Contrail ice formation, Primary soot particle sizes, Volatile particles, Sustainable aviation fuel, Lean-burning engine technology, Aviation non-CO_2_ climate impact

## Abstract

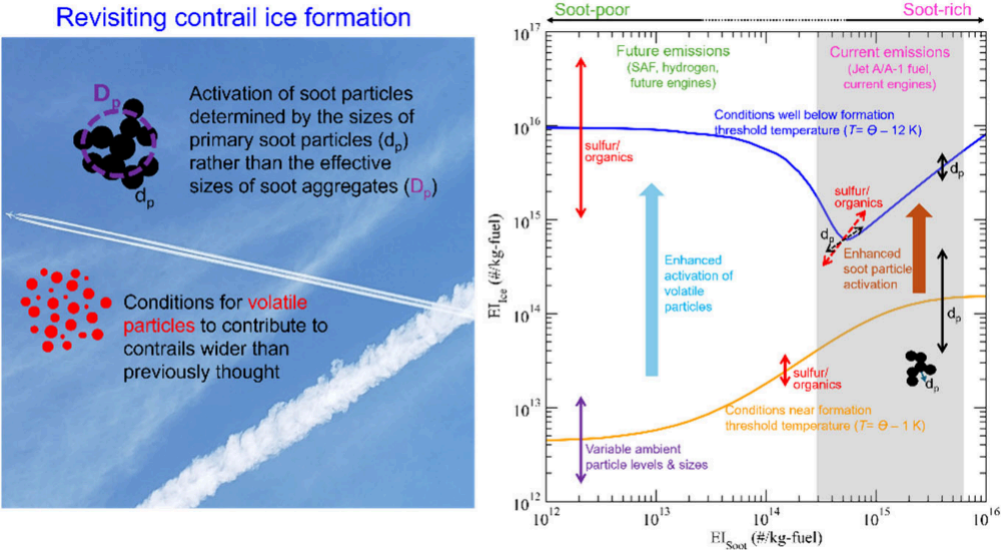

Aircraft contrails, formed largely on soot particles in current flights, are important for aviation’s non-CO_2_ climate impact. Here we show that the activation of nonvolatile soot particles during contrail formation is likely determined by the sizes of primary soot particles rather than the effective sizes of soot aggregates as assumed in previous studies, which can explain less-than-unity fractions of soot particles forming contrail ice particles as recently observed during ECLIF (Emission and CLimate Impact of alternative Fuels) campaigns. The smaller soot primary sizes compared to aggregate sizes delay the onset of contrail ice formation, increase the maximum plume supersaturation reached in the contrail plume, and thus increase the probability of small volatile particles contributing to the total contrail ice particle number. This study suggests that the range of conditions for volatile plume particles to contribute significantly to the contrail ice number budget is wider than previously thought. As the aviation industry is moving toward sustainable aviation fuel and/or lean-burning engine technology, which is expected to reduce not only the emission index of nonvolatile soot particles but also the sizes of primary soot particles, this study highlights the need to better understand how the combined changes may affect contrail formation, contribution of volatile particles, and climate impacts.

## Abstract 2

New insights into processes controlling contrail formation regarding the impact of primary soot particle sizes and volatile particles can help assess and mitigate the climate impacts of aviation emissions.

## 1 Introduction

Global aviation affects climate through the greenhouse effect of emitted CO_2_ as well as non-CO_2_ effects, including the formation of contrails and the associated impact on high-level ice clouds (cirrus).^1−4^ Global aviation contributed ∼3.5% to the anthropogenic climate forcing in 2018,^3^ and this contribution is expected to increase significantly as a result of the projected factor of ∼3.8 increase in air traffic from 2018 to 2050.^5^ About two-thirds of aviation climate forcing is currently due to non-CO_2_ effects,^3^ but there exist large uncertainties in the present assessment of aviation non-CO_2_ effects, especially impacts associated with contrail cirrus, which has a “low” level of confidence.^4^

Contrail ice crystals form when aerosol particles present in aircraft exhaust plumes activate into water droplets in water-supersaturated conditions and subsequently freeze by homogeneous nucleation.^6−11^ There are several types of particles present in exhaust plumes: besides a small number of entrained ambient aerosol particles, ultrafine liquid volatile particles (mean diameters of generally <10 nm) mainly forming on chemi-ions and composed of condensable sulfur and hydrocarbons (organics),^12−14^ larger soot particles (mean mobility diameters of few tens of nm) forming during fuel combustion,^15,16^ and lubrication oil droplets under certain conditions.^17^ Previous observations and model simulations showed that soot particles dominate contrail ice formation in current flights,^18−20^ although volatile particles can become important when fuel sulfur content (FSC) greatly exceeds average values^8,21^ or soot emissions are very low (<∼10^14^ kg-fuel^–1^).^9^

Because of the relatively high water-vapor supersaturation reached in exhaust plumes when ambient conditions favor contrail formation, nearly all soot particles are activated and subsequently freeze, despite their low hygroscopicity.^22^ Recent measurements during ECLIF (Emission and CLimate Impact of alternative Fuels) campaigns 1–3 indicated that a substantial fraction (14–52%) of nonvolatile or soot particles are not activated to form contrail ice particles, especially when sustainable aviation fuel (SAF) is used and FSC is low (<∼10 ppm).^23−25^ It is important to understand the reason(s) underlying the inactivation of some fraction of soot particles and the implications for aviation climate forcing, especially as the aviation sector is moving toward using SAF.

We hypothesize that the inconsistency between model predictions and ECLIF measurements regarding the fraction of soot particles forming contrail ice particles lies in the impact of the soot particle sizes on their activation during contrail formation. To our knowledge, all contrail models calculate soot activation using observed soot particle number size distributions (PNSDs) based on mobility diameters, or effective diameters, of soot aggregates (*D*_p_).^8−11^ However, soot particles from engine combustion are known to be aggregates of primary particles, which is clearly seen from transmission electron microscopy (TEM) images.^26,27^ Observed PNSDs of soot particles from common aircraft engines generally have median *D*_p_ ranging from 20 to 50 nm.^22,28,29^ As expected, the diameters of soot primary particles (*d*_p_) are smaller, generally in the range of 5–30 nm.^26,30,31^ The increasing biofuel content of aviation fuels is known to lower aromatic concentration and reduce the sizes of both aggregates and primary particles.^30^ Compared with *D*_p_, *d*_p_ is much closer to the sizes of volatile particles. If the activation of soot particles is indeed controlled by *d*_p_, then the conditions under which the contribution of hydrophilic volatile particles becomes important may be affected.

The simultaneous measurements of nonvolatile soot particles and ice particles under various fuel compositions (from 100% kerosene jet fuel to 100% SAF) and ambient conditions during the ECLIF missions provide a new opportunity to revisit and refine our understanding of contrail ice particle formation and controlling parameters. In this study, we use an updated aerosol and contrail microphysics model to investigate the contrail ice formation process and its controlling factors observed during ECLIF1–3, focusing on partial soot activation and the yet unexplored impact of primary soot sizes, as well as its implications for volatile particles’ contribution of contrail ice formation.

## 2 Methods

### 2.1 ECLIF (Emission and CLimate Impact of alternative Fuels) Campaigns 1–3

The ECLIF campaigns evaluated the effects of using alternative fuels and clean-burning jet engines on aircraft particle emissions and, in turn, the links between these emissions and contrail properties.^24,25,32^ Both ECLIF1 (2015) and ECLIF2 (2018) were performed in Germany and used DLR A320 Advanced Technology Research Aircraft (ATRA) equipped with IAE V2527-A5 engines as the source aircraft. ECLIF3 (2021) was conducted over southern France and nearby ocean regions and used an Airbus A350-941 with Rolls-Royce Trent XWB-84 engines as the emission source aircraft.^25^ During ECLIF1 and ECLIF3, the DLR Falcon 20-E5 research aircraft was used to probe plume composition and thermodynamic properties,^25,32^ whereas NASA’s DC-8 aircraft was used for that purpose during ECLIF2.^24^

All ECLIF in-flight measurements have been detailed in previous publications.^24,25,32^ Here we focus on the model interpretation of contrail measurements under six conditions obtained during these campaigns. Table 1 gives relevant information about aircraft type, fuel composition, and ambient conditions as well as simultaneously measured emission indices of nonvolatile soot particles (EI_soot_) and contrail ice particles (EI_ice_) for the six cases. Most of the measured values in Table 1 are from Voigt et al.^24^ for ECLIF1–2 and Märkl et al.^25^ for ECLIF3 and are averages over the sampling periods given. Voigt et al.^24^ mentioned FSC < 10 ppmm for Case 4, and the value of 4.1 ppmm for this case is from Jones and Miake-Lye.^33^ The emission index of water vapor (EI_H_2_O_) is calculated from the fuel hydrogen content. The plume ages at the points of measurement range from 39 to 142 s.^24,25^ RH_ice_ given in Table 1 represents average values during the duration of the measurements for each case when RH_ice_ > 100%. The fraction of soot particles activated into water droplets and forming contrail ice particles (*F*_ice_) is calculated as EI_ice_/EI_soot_, and the compound uncertainty for *F*_ice_ is also shown. While there are large uncertainties, all measurements consistently indicate that *F*_ice_ is less than unity, especially for Cases 4 and 6, where FSC is very low.

**Table 1:** Ambient and Aircraft Conditions and Measurements for the Six Case Studies of ECLIF Campaigns Reported in This Work^a^

	Cases					
	1	2	3	4	5	6
	ECLIF1	ECLIF1	ECLIF2	ECLIF2	ECLIF3	ECLIF3
Source aircraft	Airbus A320	Airbus A320	Airbus A320	Airbus A320	Airbus A350	Airbus A350
Fuel	100% Jet A-1	59% JetA-1 + 41% FT-SPK	51% JetA-1 + 49% HEFA-SPK	70% JetA-1 + 30% HEFA-SPK	100% Jet A-1	100% HEFA-SPK
*H* (km)	10.67	10.364	9.726	9.656	10.626	10.621
*T*_amb_ (K)	215	220	218	216	213.3	213.8
RH_ice_ (%)	120	111.5	120	110	108	107.5
Contrail age (s)	39–132	48–134	53–140	41–116	104–142	73–92
Sampling time (s)	482	280	284	119	183	123
*V*_plane_ (km/h)	802.75	716.3	938.6	938.6	1044.81	1052.22
FFR (kg/h)	1180	820	1132	1091	2700	2751.3
FSC (ppm)	1350	570	70	4.1	211	7
EI_H_2_O_ (kg/kg-fuel)	1.227	1.283	1.287	1.297	1.258	1.35
EI_soot_ (10^15^ #/kg-fuel)	4.9 ± 0.6	2.5 ± 0.2	2.7 ± 0.6	2.3 ± 0.6	0.95 ± 0.3	0.61 ± 0.07
EI_ice_ (10^15^ #/kg-fuel)	4.2 ± 0.6	2 ± 0.2	2.3 ± 0.2	1.1 ± 0.4	0.78 ± 0.4	0.34 ± 0.15
*F*_ice_	0.86 ± 0.23	0.80 ± 0.14	0.85 ± 0.26	0.48 ± 0.30	0.82 ± 0.68	0.56 ± 0.31

a Data mostly from Voigt et al.^24^ and Märkl et al.^25^*H* is flight altitude, *T*_amb_ is ambient temperature, RH_ice_ is relative humidity with respect to ice, *V*_plane_ is plane flight speed, FFR is fuel flow rate, and FSC is fuel sulfur content. FFR values for ECLIF1–2 are measured data from Voigt et al.^24^ while ECLIF3 FFR for Jet A-1 has been estimated from the information given in www.airliners.net and then the HEFA FFR was derived from this estimate by adding +1.9% as indicated in Märkl et al.^25^

### 2.2 An Updated Aerosol and Contrail Microphysics (ACM) Model

The ACM model used here is a parcel model of jet plume aerosol and ice microphysics developed in the late 1990s.^8,12,34^ Nonvolatile (soot) and volatile particles (aqueous solution droplets) are discretized over a particle size grid that can be modified as needed. The model employs a kinetic approach to simulate the formation and growth of volatile particles, and the effects of electrical charge are considered. Some algorithms and thermodynamic data used in the kinetic nucleation model were improved in the past two decades^35,36^ and are incorporated in the volatile particle formation portion of the current ACM model, as detailed in a recent publication.^37^

Turbulent plume mixing with ambient air cools the exhaust plume, entrains ambient particles and H_2_O, and dilutes the plume constituents. Uniform mixing is assumed across the plume cross-section, representing average conditions. During the early evolution of an aircraft exhaust plume, volatile particles are formed when sulfuric acid vapor (oxidized from a small fraction of sulfur in the fuel during combustion) becomes sufficiently supersaturated, while the hygroscopicity of soot particles is modified through the collection of sulfuric acid (or other condensable species) on the carbon surfaces. The model keeps track of the amount of aqueous sulfuric acid coating on soot particles via condensation of sulfuric acid as well as the coagulation scavenging of volatile particles by soot aggregates. As exhaust gases continue to cool through isobaric mixing with colder ambient air, water droplets form by activation of plume particles if the mixture reaches saturation with respect to supercooled liquid water. The minimum dry diameter (*D*_dry_) of aerosol particles that can be activated at a given water supersaturation ratio (*S*) is determined according to the κ-Köhler theory that considers both Kevin and solution effects,^38^
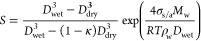
1where κ is the hygroscopicity parameter, *ρ*_w_ is the density of water, *M*_w_ is the molecular weight of water, σ_s/a_ is the surface tension of the solution/air interface, *R* is the universal gas constant, *T* is temperature, and *D*_wet_ is the wet diameter of particles in equilibrium with water vapor. For pure nonvolatile hydrophobic soot particles, κ = 0 and *D*_wet_ = *D*_dry_. The κ value of soot particles coated with soluble species (or other mixed particles) is given by the simple mixing rule,^38^
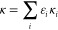
2where *ε*_*i*_ and *κ*_*i*_ are the dry volume fraction and hygroscopicity parameter of individual component *i* in the mixture, respectively.

In fresh aircraft plumes before the onset of contrails (*t* < ∼0.1 s), the volume of coated volatile species is generally much less than the soot volume (i.e., *ε*_volatile_ ≪ *ε*_soot_), *κ* ≪ 1, and *D*_wet_ is close to *D*_dry_. Under these conditions, the activation of coated soot particles is dominated by the Kelvin or curvature effect, i.e., the exponent term in eq 1. As pointed out in the Introduction, the soot particles from engine combustion are known to be aggregates of primary particles, and physically, the curvature (or Kelvin) effect should be determined by the diameters of soot primary particles (*d*_p_) instead of the effective diameters of the aggregates (*D*_p_). As a result, for soot aggregates, the κ-Köhler equation (eq 1) becomes
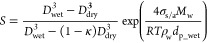
3where *d*_p_wet_ is the wet diameter of soot primary particles and is close to *d*_p_ when the volume of coated volatile species is much less than the soot volume.

When *S* > 1, the number of droplets formed depends on the number concentration of particles (both nonvolatile and volatile) that can be activated into droplets, which is a function of *S* and the size distribution and hygroscopicity of both soot and volatile aerosols (owing to the Köhler effect). Further, water droplets can freeze and continue to grow at the expense of the remaining liquid droplets when the relative humidity is below the saturation level over liquid water but above that over ice. Due to the low temperatures at which contrails form (typically <225 K), homogeneous freezing rates are very large. The associated rate coefficient used in the ACM is extrapolated from experimental data.^39^

In the ACM model, a hybrid bin structure is adopted, with the core component being soot and/or volatiles. The current ACM model employs 150 bins and covers dry diameters from 0.55 nm (diameter of one H_2_SO_4_ molecule) to 15 μm, with higher resolution for particles in the size range of 0.55–80 nm (100 bins), where most soot and volatile particles are located. The water content of the inactivated aerosols is calculated by assuming instantaneous water vapor equilibrium with the plume environment, while the water content of the activated droplets is determined by integrating over time the net kinetic flux of water vapor to/from the particle surface. Soot, volatile, and ambient particles comprise the three basic types of aerosols currently treated in the model. With ion effects also considered, we explicitly solve for the evolution of volatile particles’ charged and neutral populations. Moreover, if a contrail forms, we keep track of both liquid and ice particles and quantify their competition for water vapor (calculating the shift of water from the liquid to the ice phase, as occurs in mixed-phase clouds). Coalescence between inactivated aerosols and activated (liquid and ice) particles is also explicitly treated.

Using the ACM model, we simulated contrail formation for six different cases (or conditions) observed during ECLIF campaigns 1–3 (Table 1). In addition to ambient conditions and aircraft operation/emission data given in Table 1, the ACM model needs additional information to simulate particle and contrail ice formation and evolution, including the plume dilution ratio, size distributions of soot and ambient particles, and sulfur-to-sulfuric acid conversion fraction. In the simulations described in Section 3, the average dilution ratio as a function of plume age is based on the parametrization derived by Schumann et al.^40^ from aircraft exhaust dilution measured in more than 70 plume encounters in the upper troposphere and lower stratosphere for plume ages of milliseconds to 95 min, for a wide range of aircraft including medium-sized (e.g., B727) and wide-body aircraft (mostly B747). The size distribution of ambient particles entrained into the plume is assumed to have two log-normal modes with number concentration, dry median diameter, and standard deviation of 1000 #/cm^3^, 10 nm, 1.6 and 10 #/cm^3^, 150 nm, 1.6, respectively, the same as what was assumed in the study by Kärcher and Yu^9^ (named KY09 thereafter). The percentage of fuel sulfur converted to sulfuric acid (*S*_conv_) during combustion is generally between 2 and 4%^14,41,42^ and is assumed to be 3%, the same as in KY09. The model time step (d*t*) changes with plume age (considering the concentration change of gaseous and particulate species due to dilution). d*t* starts at 10^–5^ s at plume age *t*_0_ = 0.003 s and gradually increases to 10^–3^ s at plume age *t* = 0.1 s, 1.5 × 10^–3^ s at *t* = 0.15 s, and 0.01 s at *t* = 1 s, enabling the detailed microphysics (including aerosol formation and growth, dilution and RH peaks, and contrail formation) to be well resolved.

Our plume simulations cover plume ages up to 147 s. Since all the six ECLIF cases studied here have RH_ice_ > 100% and ambient temperature ∼6–13 K below the contrail formation threshold temperature, we expect the effect of plume dynamics to be relatively small.

### 2.3 Diameters of Soot Primary Particles versus Aggregates

The sizes of soot primary particles and aggregates from aircraft engines depend on engine types, operation conditions, and fuel compositions.^26,30,31^ The size distribution of soot particles (aggregates) for all six cases is assumed to be log-normal, with a median diameter (*D*_p_) of 35 nm and standard deviation of 1.6, which is based on the ground-based characterization of PNSDs in the framework of the ECLIF campaign using an Airbus A320 (V2527-A5 engines).^29^ The sizes of soot particles from more modern Airbus A350 engines are expected to be smaller than those of Airbus A320 engines, but direct measurements are not yet available in the literature.

TEM measurements indicate that larger aggregates tend to contain larger primary particles.^26,30,31^ This is clearly seen in the scattering plot of *d*_p_ versus *D*_p_ given in the Supporting Information (Figure S1a). While *d*_p_ generally increases with *D*_p_, the ratios of XR = *d*_p_/*D*_p_ are relatively more constant (a weaker inverse dependence on *D*_p_) (Figure S1b). For the data shown in Figure S1, XR has a median value of 0.41, with large variations of values ranging from 0.17 to 0.73. As mentioned earlier, all previous contrail formation modeling studies calculate the activation of soot particles using aggregate sizes (*D*_p_), which is equivalent to XR = 1 here. It should be noted that a more sophisticated parametrization of the relationship between *d*_p_ and *D*_p_ has been derived previously based on limited measurements.^43,44^ In the present study, we use a simplified average XR value derived from a large set of measurements (including various engine types, operation settings, and fuels) and carry out sensitivity studies using a range of XR values indicated from these measurements.

Here we use XR = 0.41 as the baseline case and carry out sensitivity studies to explore the impacts of soot primary sizes. The impact of variations in XR can also be interpreted as the equivalent effect of changes in *D*_p_, which is relevant especially as increasing biofuel content of aviation fuels is known to lower aromatic concentration and reduce the sizes of both aggregates and primary particles.^30^ By varying XR, we can also assess the potential effects of the likely smaller *D*_p_ of Airbus A350 engines used in ECLIF3 compared with those assumed here based on the ground-based characterization of soot particles for Airbus A320 used in ECLIF1 and 2. In the sensitivity study reported in Section 3, we vary XR from 0.1 to 1, taking into account potential changes in both primary and aggregate sizes associated with future aviation fuels or more modern engines, as well as the fact that previous contrail studies used *D*_p_ to calculate soot activation.

## 3 Results and Discussion

**Figure 1:**
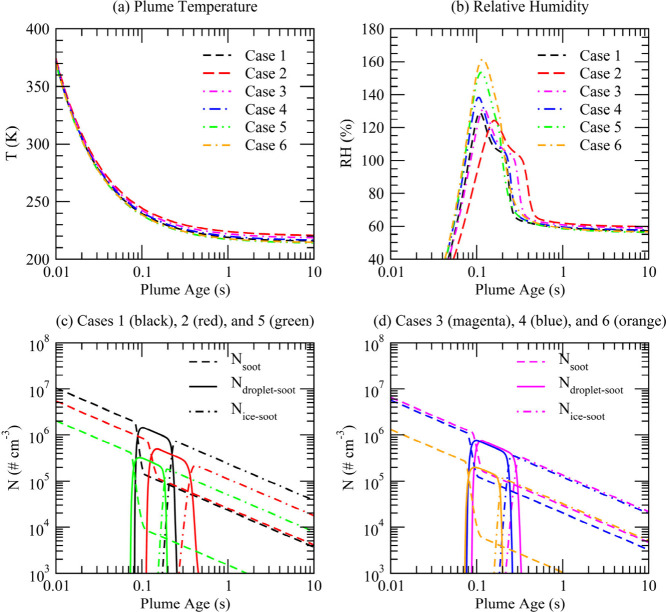
Evolutions of (a) temperature (*T*), (b) relative humidity (RH) (with respect to liquid water), and (c, d) number concentrations of unactivated soot particles (*N*_soot_, dashed lines), water droplets formed on activated soot particles (*N*_droplet-soot_, solid lines), and contrail ice particles formed on soot particles (*N*_ice-soot_, dot-dashed lines) in the plumes corresponding to the six cases (Table 1) observed during ECLIF1–3. A baseline *d*_p_-to-*D*_p_ ratio (XR) of 0.41 is assumed in the model simulation.

Figure 1 shows the evolution of temperature (*T*), relative humidity (RH), and number concentrations of soot particles (*N*_soot_), water droplets formed on activated soot particles (*N*_droplet-soot_), and contrail ice particles formed on soot particles (*N*_ice-soot_) in the plumes of the six contrail formation cases observed during ECLIF1–3. In all six cases, because of relatively high EI_soot_, the contrail particles were dominated by soot particles, and contributions of plume volatile and entrained ambient particles were negligible. Therefore, only soot particles and droplets/ice particles formed on soot are shown in Figure 1c,d. For the simulation shown in Figure 1, a baseline XR value of 0.41 is assumed. As a result of the rapid dilution, the exhaust plume *T* decreases quickly and approaches that of ambient air within seconds (Figure 1a). The RH in the young plume (*t* < ∼0.5 s) is determined by EI_H_2_O_ and plume *T* as well as the condensation of water vapor on activated and/or frozen particles after RH_water_ exceeds 100%. Due to the difference in *T*_amb_ (see Table 1) and thus *T* in the plume as well as EI_H_2_O_, the plume age when RH_water_ reaches 100% differs slightly, ranging from 0.07 s for Case 6 to 0.11 s for Case 2 (Figure 1b). Thereafter, some of the large soot particles are activated into liquid droplets (Figure 1c,d, noting the decrease in *N*_soot_ and the increase in *N*_droplet-soot_). When RH_water_ reaches 100%, the plume *T* is ∼242–247 K, too high for the droplets to freeze homogeneously.

As the plume continues to dilute and cool, RH continues to increase, and more particles are activated. When the RH increase due to dilutional cooling is balanced by the condensation of water vapor on activated particles, RH reaches its maximum at plume ages of around 0.1–0.15 s (Figure 1b), with RH_max_ values depending on ambient *T* (and RH), dilution rate, EI_H_2_O_, and the number of particles available for activation into droplets. RH_max_, which determines the smallest particles that can be activated and hence the maximum *N*_droplet-soot_, is lowest for Case 2 (124.2% at *t* = 0.160 s) and highest for Case 6 (161.5% at *t* = 0.117 s). Shortly after RH reaches its maximum and as the plume approaches the homogeneous freezing temperature, activated droplets start freezing into ice particles (Figure 1c,d, noting the quick decrease of *N*_droplet-soot_ and rapid increase of *N*_ice-soot_). Thereafter, RH drops quickly due to condensation on ice particles and approaches ambient RH level at plume ages of ∼1 s. Since all six cases have RH_ice_ > 100% (Table 1), contrail ice particles persist, although their number concentrations decrease with increasing plume ages due to dilution (Figure 1c,d).

**Figure 2:**
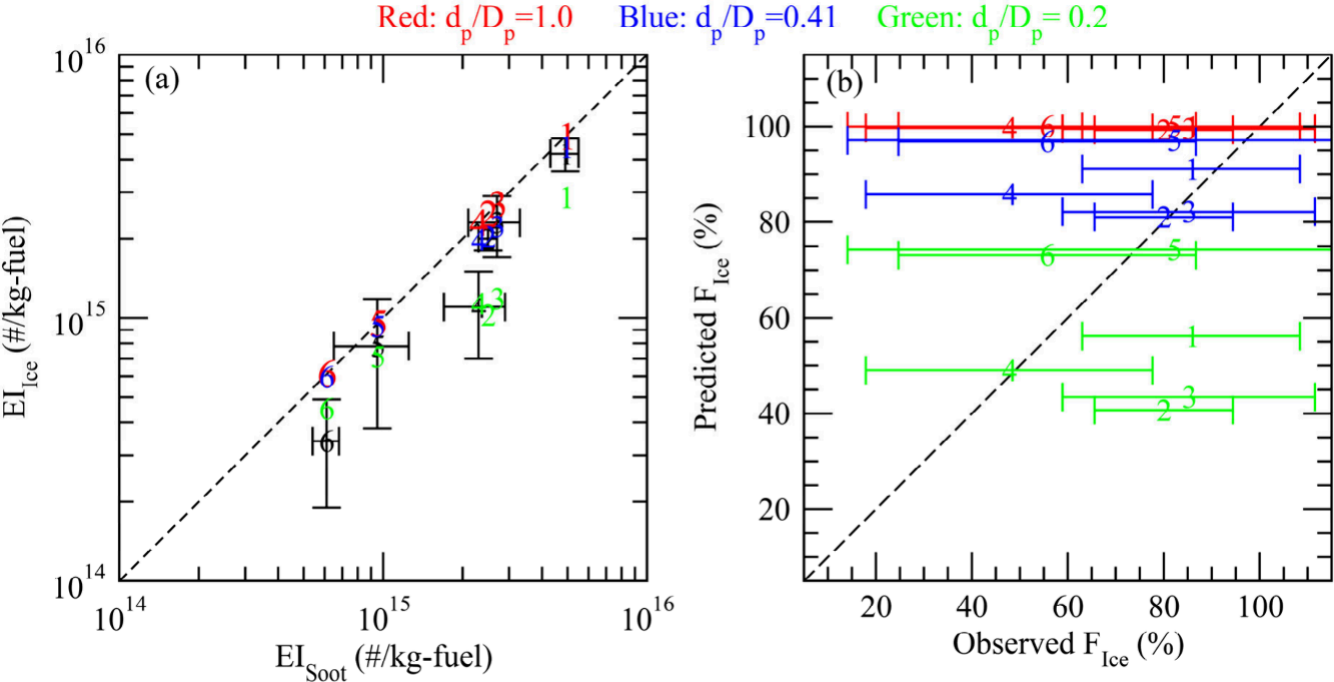
(a) Predicted versus observed EI_ice_ as a function of the observed EI_soot_, and (b) predicted versus observed *F*_ice_ for the six ECLIF1–3 cases under three assumed *d*_p_-to-*D*_p_ ratios: 1.0 (red), 0.41 (blue), and 0.2 (green). The number shown corresponds to Case # in Table 1, and the measurement uncertainties are indicated with error bars. In (a), the black numbers are the observed values.

**Figure 3:**
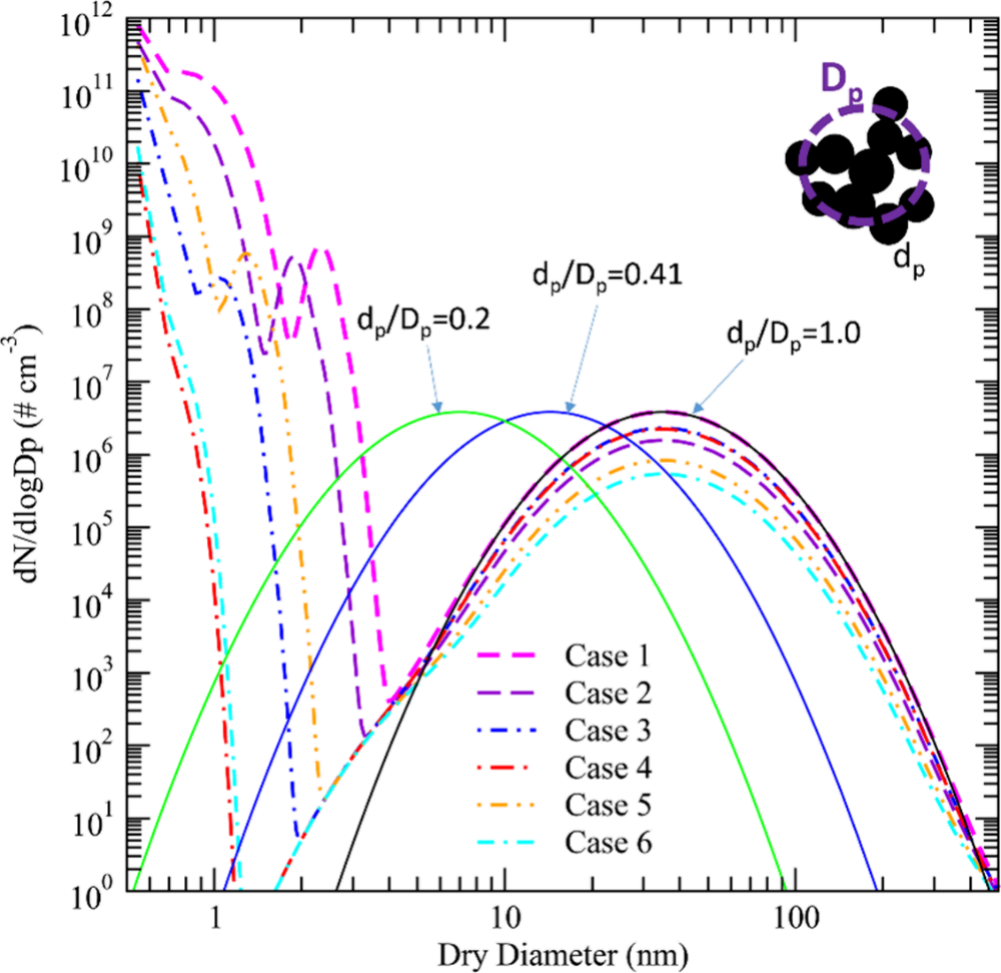
Particle size distributions right before the relative humidity in the plume reaches 100% for the six study cases given in Table 1. The inset is a schematic drawing of soot primary and aggregates to illustrate diameters of aggregates (*D*_p_) and primary soot particles (*d*_p_). The dashed lines are the size distributions of both volatile particles and soot aggregates. The three solid lines show the (dry) size distributions of the primary particles within aggregates for XR (=*d*_p_/*D*_p_) of 0.2, 0.41, and 1.

Because of the high supersaturation ratios reached (RH over water up to 120%–160%) in the plume for all six cases (Figure 1b), the model simulations show that most of the soot particles activated into water droplets froze to form contrail ice particles (Figure 1c,d). While this is consistent (within the uncertainty) with the observed *F*_ice_ for Cases 1–3 and 5 as shown in Table 1, it is not for Cases 4 and 6. To illustrate this more clearly, Figure 2 compares predicted and observed EI_ice_ as functions of the observed EI_soot_ as well as predicted *F*_ice_ versus observed *F*_ice_ for the six cases under three assumed XR (=*d*_p_/*D*_p_) values (0.2, 0.41, and 1.0). The particle size distributions right before RH_water_ reaches 100% are given in Figure 3. Figure 2a shows that the model generally captures the dependence of EI_ice_ on EI_soot_, i.e., the higher EI_soot_ is, the larger is EI_ice_. However, when the activation of soot particles is calculated based on their effective aggregate sizes (as commonly done in contrail model simulations), the model indicates almost 100% soot activation (see numbers in red in Figure 2) and tends to overpredict EI_ice_ for all cases. While there exist uncertainties, the simultaneous measurements of soot and ice particles indicate less than unity activation of soot particles, and *F*_ice_ drops to ∼50% for the two cases with extremely low FSC (Cases 4 and 6) (Table 1 and Figure 2b). The activation of soot particles assuming XR = 0.41 (blue numbers in Figure 2, and blue solid line in Figure 3) reduces the model predicted *F*_ice_ by ∼9–20% (absolute value, i.e., from ∼100% to 91–80%) for Cases 1–4, enabling a near perfect agreement with measured values for Cases 1–3 and better agreement for Case 4. For Cases 5 and 6, *F*_ice_ decreases by only a few percent when XR = 0.41 is assumed instead of using aggregate sizes (i.e., XR = 1.0), mainly due to colder ambient *T* (Table 1) and much higher RH_max_ (Figure 2b). If XR is assumed to be 0.2 (green solid line in Figure 3), *F*_ice_ drops significantly for all cases by ∼20–40%, making *F*_ice_ for Cases 4–6 in much better agreement with measured values but *F*_ice_ for Cases 1–3 much lower than the observed values. Clearly, the primary particle size can influence the fraction of soot particles forming contrail ice particles, and such an influence varies with engine emissions and ambient conditions.

Figure 3 shows that high concentrations of volatile particles (emission index in the order of 10^17^ kg-fuel^–1^) are formed for ECLIF Cases 1–3 and 5 (on chemi-ions, noting the bimodal size distributions of volatile particles for cases with relatively higher FSC) but their sizes are too small (dry diameter <4 nm) to contribute to the contrail ice particles. For Cases 4 and 6, because of the extremely low FSC (<10 ppm), the volatile particles formed are smaller than 1.5 nm. It should be noted that the contribution of organic species to volatile particle nucleation and growth is not considered in the present simulation due to the lack of information about speciation and concentrations of such organic species, and thus, the sizes of volatile particles given in Figure 3 are likely lower limit values, underestimating the impact of volatile plume particles to contrail ice formation. The difference in volatile-particle sizes is mainly caused by the variation in FSC, with some of the volatile particles growing larger than 2.5 nm for Cases 1 (FSC = 1350 ppm) and 2 (FSC = 570 ppm). Although we assume that S_conv_ = 3.0%, the variations or uncertainties in *S*_conv_ also affect the sizes of the volatile particles.

**Figure 4:**
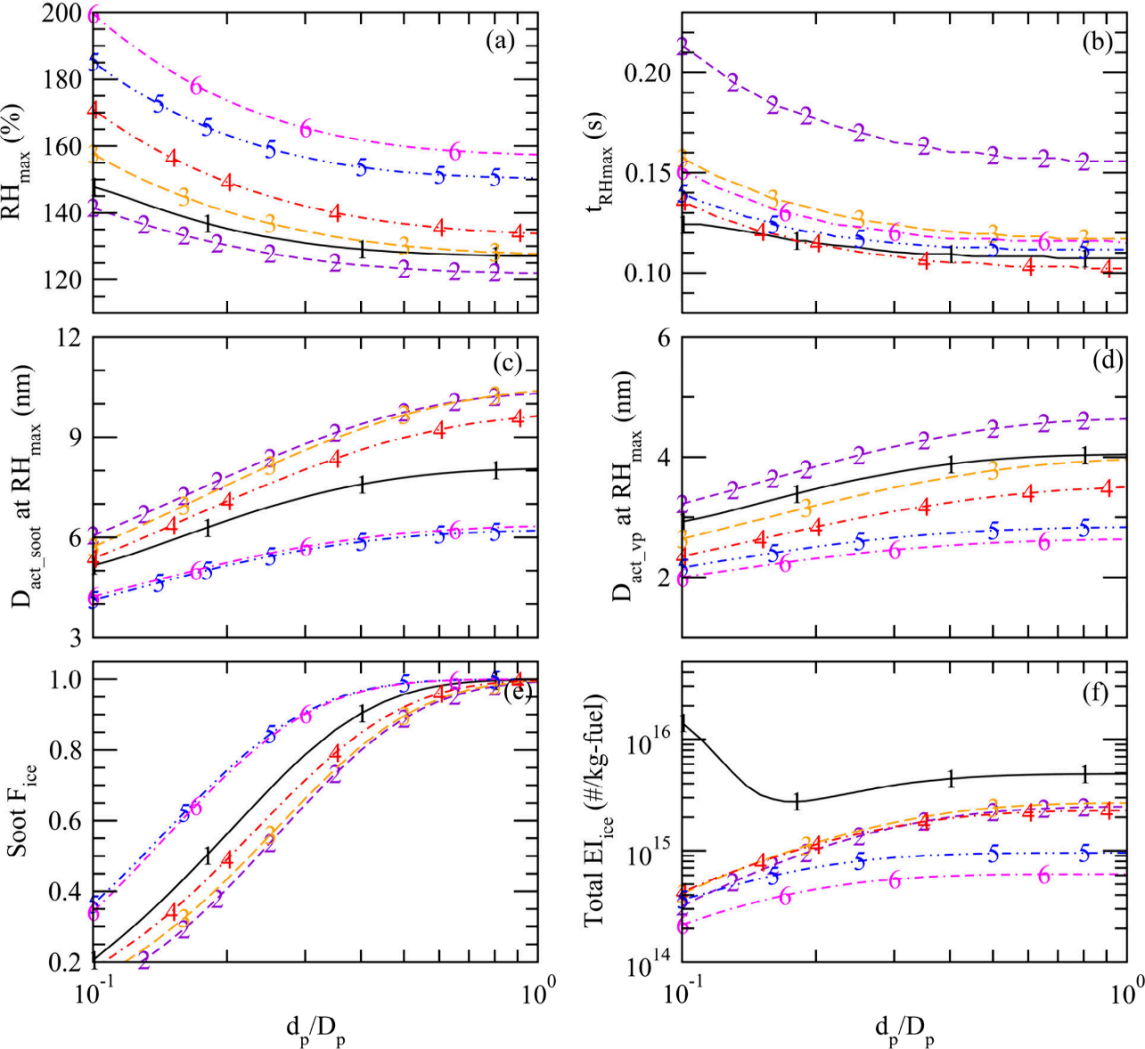
Effects of primary soot particle diameter (*d*_p_) on (a) maximum RH reached in the plume (RH_max_), (b) plume age of RH_max_, activation diameter at RH_max_ for (c) soot particles (*D*_act_soot_) and (d) volatile particles (*D*_act_vp_), (e) fraction of soot particles forming ice particles (*F*_ice_), and (f) total EI_ice_.

To understand how *d*_p_ influences the fraction of soot particles forming contrail ice particles and the potential activation of volatile particles, we carry out sensitivity studies for all six ECLIF cases by varying XR (=*d*_p_/*D*_p_) from 0.1 to 1.0. Figure 4 shows the effect of XR (or *d*_p_) change on RH_max_ (over water), plume age at RH_max_, activation (dry) diameter of both volatile and nonvolatile (soot) particles at RH_max_, *F*_ice_soot_, and total EI_ice_ for the six observation cases (Table 1). RH_max_ determines the smallest size of particles that can be activated and thus the number concentration of contrail ice particles, which is important for contrail cirrus radiative forcing.^45^ RH_max_ is controlled by the balance between the source (EI_H_2_O_ and plume cooling rate) and sink (condensation and dilution) terms. As XR (or *d*_p_) decreases, the water supersaturation ratio needed to activate the soot particles increases because of the Kelvin effect. As a result, the value of RH_max_ increases (Figure 4a) and the plume age when RH reaches its maximum (*t*_RH_max__) increases (Figure 4b). In addition to *d*_p_, *t*_RH_max__ and RH_max_ are influenced by ambient *T*, dilution rate, EI_H_2_O_, EI_soot_, and FSC as well. Case 2 has the lowest RH_max_ (∼125% when XR > 0.5 and >∼130% when XR < 0.2) and reaches RH_max_ the latest (*t*_RH_max__ ≈ 0.16–0.21 s), while Case 6 has the highest RH_max_ (∼157% when XR = 1.0 and 200% when XR = 0.1), and Cases 1, 4, and 5 reach RH_max_ fastest with *t*_RH_max__ ranging from 0.1 to 0.14 s for the ranges of XR considered.

The values of RH_max_ determine the minimum activation sizes of volatile (*D*_act_vp_) and nonvolatile soot (*D*_act_soot_) particles, which are given in Figure 4c,d. The corresponding fractions of soot particles forming contrail ice particles (*F*_ice_) and total EI_ice_ (ice forming on both soot and volatile particles) are provided in Figure 4e,f. In calculating *D*_act_soot_, the model considers the amount of sulfuric acid coated on soot particles and, hence, the hygroscopicity change, which depends on FSC and *S*_conv_. Because of the increase in RH_max_ as XR decreases, both *D*_act_soot_ and *D*_act_vp_ decrease. It is interesting to note that the effect of XR is relatively small when XR > ∼0.5 (corresponding to median *d*_p_ of ∼17 nm) and is larger when XR < ∼0.5. This is because when XR > ∼0.5, under realistic atmospheric conditions and engine emission scenarios as in ECLIF1–3 campaigns, nearly all soot particles are activated and form contrail particles (see Figure 4e), and thus RH_max_ is no longer sensitive to XR (Figure 4a). In contrast, when XR < ∼0.5, all parameters shown in Figure 4 are sensitive to XR. Since XR is generally smaller than 0.5 (see Figure S1), our finding that the activation of soot particles depends on *d*_p_ instead of sizes of aggregates and the large sensitivity of soot activation to the sizes of primary soot particles smaller than ∼17 nm (i.e., XR < ∼0.5) provides a possible explanation for the consistently less-than-unity *F*_ice_ values observed during ECLIF 1–3 (also see Figure 3 and Table 1).

The use of SAF reduces not only the emission index (or number concentration) of nonvolatile soot particles but also the sizes of the associated primary soot particles.^30^ It is therefore important to understand how the combined changes may affect contrail formation and climate impacts. Both a reduction in EI_soot_ and a decrease in *d*_p_ associated with SAF or other technology (such as lean-burning engines^31^) will reduce the contribution of soot particles to the total number of contrail ice particles. However, as first shown by KY09, volatile particles may become important when EI_soot_ is small (<∼10^13^–10^14^ kg-fuel^–1^). The present study shows that, in addition to EI_soot_ reduction, a decrease in *d*_p_ can also enhance the contribution of volatile particles to contrail ice particle formation. This is illustrated in Figure 4d, where a smaller *d*_p_ leads to smaller sizes of volatile particles that are activated, down to 2–3 nm when XR = 0.1. Figure 3 shows that ion-mode particles can grow above 2 nm for all cases except the two with extremely low FSC (i.e., Cases 4 and 6). Figure 4f shows that when XR is small enough (<∼0.15), some of the volatile particles for Case 1 are large enough to be activated and significantly increase EI_ice_, well above the contribution of soot particles.

This study does not consider the contribution of condensable organics to volatile particles and thus represents the minimum sizes of volatile particles formed in the plume. The real contribution of volatile particles to contrail ice particle formation when *d*_p_ is small (such as in the case of 100% SAF^30^) depends on FSC (and *S*_conv_) as well as the concentrations of condensable organic species in the exhaust plume, which remain to be characterized.

**Figure 5:**
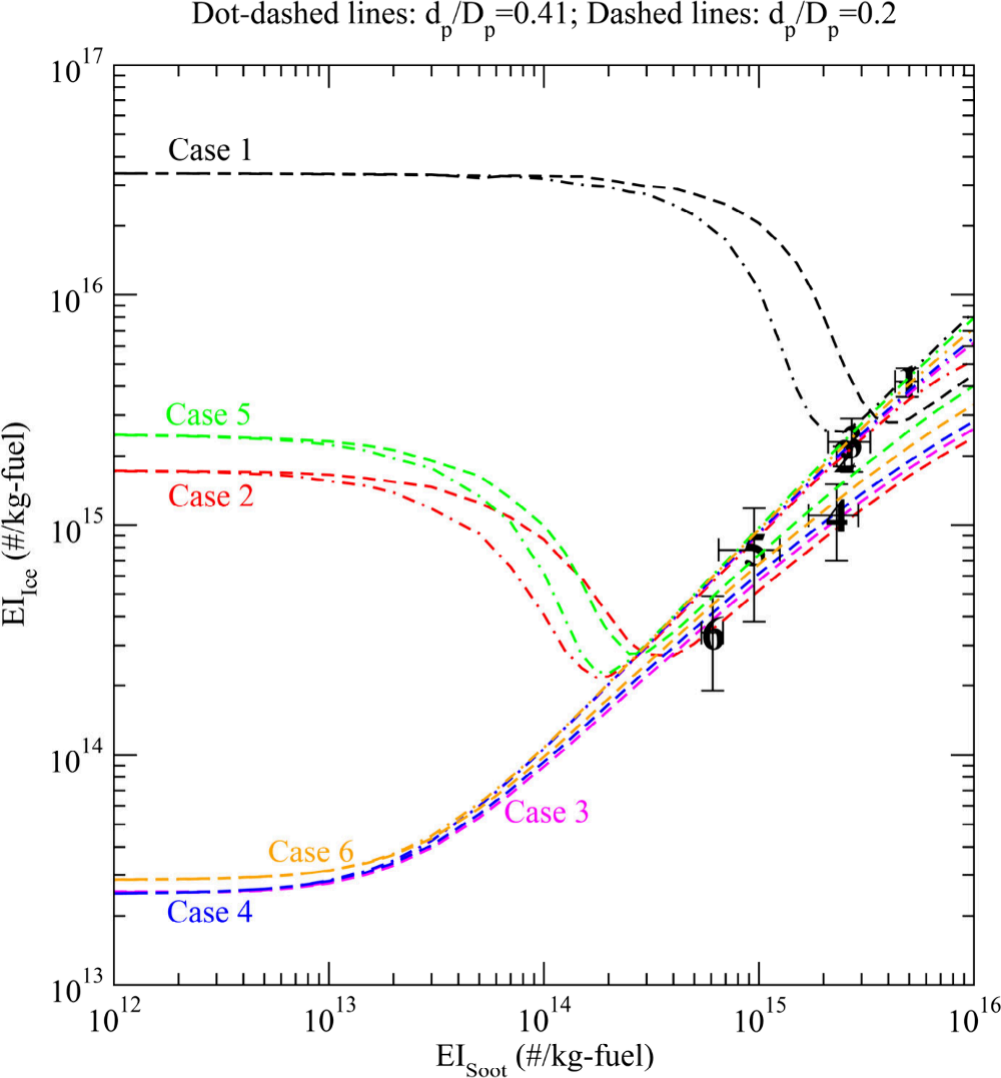
Dependence of apparent contrail EI_ice_ on EI_soot_ for six ECLIF cases at two assumed ratios of soot primary sizes (*d*_p_) to aggregate sizes (*D*_p_) (dot-dashed lines: *d*_p_/*D*_p_ = 0.41; and dashed lines: *d*_p_/*D*_p_ = 0.2). The symbols are measurements from ECLIF1–3 (Table 1).

The results shown in Figure 4 assume a constant EI_soot_. In reality, the decrease in *d*_p_ is likely to be accompanied by a simultaneous reduction in EI_soot_.^30^ To explore how simultaneous reduction in EI_soot_ and *d*_p_ may affect EI_ice_, we carried out a sensitivity study (Figure 5) by varying EI_soot_ at two fixed XR values (0.41 and 0.2) for all six ECLIF cases. We also added in Figure 5 the observed EI_ice_ versus EI_soot_ for all six cases. Note that EI_soot_ has a strong effect on EI_ice_ and, as reported in KY09, EI_ice_ may increase with decreasing EI_soot_ at certain ranges of EI_soot_ because of the contribution of volatile particles. The contribution of volatile particles depends on the size of the volatile particles and the maximum water supersaturation reached in the plume. For Cases 3, 4, and 6, the volatile particles (Figure 3) are too small to be activated under the maximum supersaturation reached in the plume (Figure 1b).

Compared with previous EI_ice_ versus EI_soot_ results described in KY09 (and later re-illustrated in a review paper by Kärcher^2^), Figure 5 represents updated simulations using the improved ACM model (Section 2.2) and reveals a number of new or different features. First, in the low-soot regime, the contributions of volatile particles can become important at temperatures substantially warmer (not far from the contrail formation threshold temperature; see Case 2, which has *T*_amb_ = 220 K) than those indicated in KY09 (*T*_amb_ = ∼213 K, well below the formation threshold temperature). This has important implications, as volatile particles can become important under ambient conditions broader than previously suggested. Second, the volatile particles can become important at EI_soot_ values higher than those indicated in KY09, especially when *d*_p_ is smaller. Under some conditions, especially when FSC is high (see curves for Case 1), volatile particles become important even in the soot-rich regime (EI_soot_ > ∼5 × 10^14^ kg-fuel^–1^). Third, EI_ice_ levels off (i.e., reaching a plateau) at low EI_soot_, which was not seen in the results of KY09. Fourth, both FSC and *T*_amb_ strongly affect the contribution of volatile particles to contrail ice formation, while KY09 focused on *T*_amb_ only. These differences between the present work and KY09 are caused by multiple factors, including different thermodynamics for volatile particle formation and updated schemes for soot activation, FSC, soot particle size distributions, and ambient conditions. Note that this study is constrained by ELCIF1–3 in situ measurements (see Table 1), whereas KY09 explored hypothetical (but typical) conditions, and the present model captures the observed dependence of EI_ice_ on EI_soot_. We consider results provided by the improved model as an upgrade, and thus they supersede our earlier EI_ice_ versus EI_soot_ predictions; however, measurements in the low-soot regime are needed to evaluate these predictions.

In addition to these new insights, we show for the first time in Figure 5 a large effect of *d*_p_ on EI_ice_ for intermediate to soot-rich values of EI_soot_ > ∼10^14^ kg-fuel^–1^. When EI_soot_ is larger than a certain threshold value (the EI_soot_ for minimum EI_ice_), which depends on FSC, *T*_amb_, and *d*_p_, EI_ice_ increases with increasing *d*_p_ because of the Kelvin effect on the activation of soot particles, with differences reaching up to a factor of ∼2 for the six cases presented when *d*_p_/*D*_p_ changes from 0.41 to 0.2. When EI_soot_ is smaller than this threshold value, EI_ice_ increases with decreasing *d*_p_/*D*_p_ because a smaller *d*_p_ inhibits the activation of soot particles and thus allows a larger RH_max_ (Figure 4a) to activate volatile particles.

**Figure 6:**
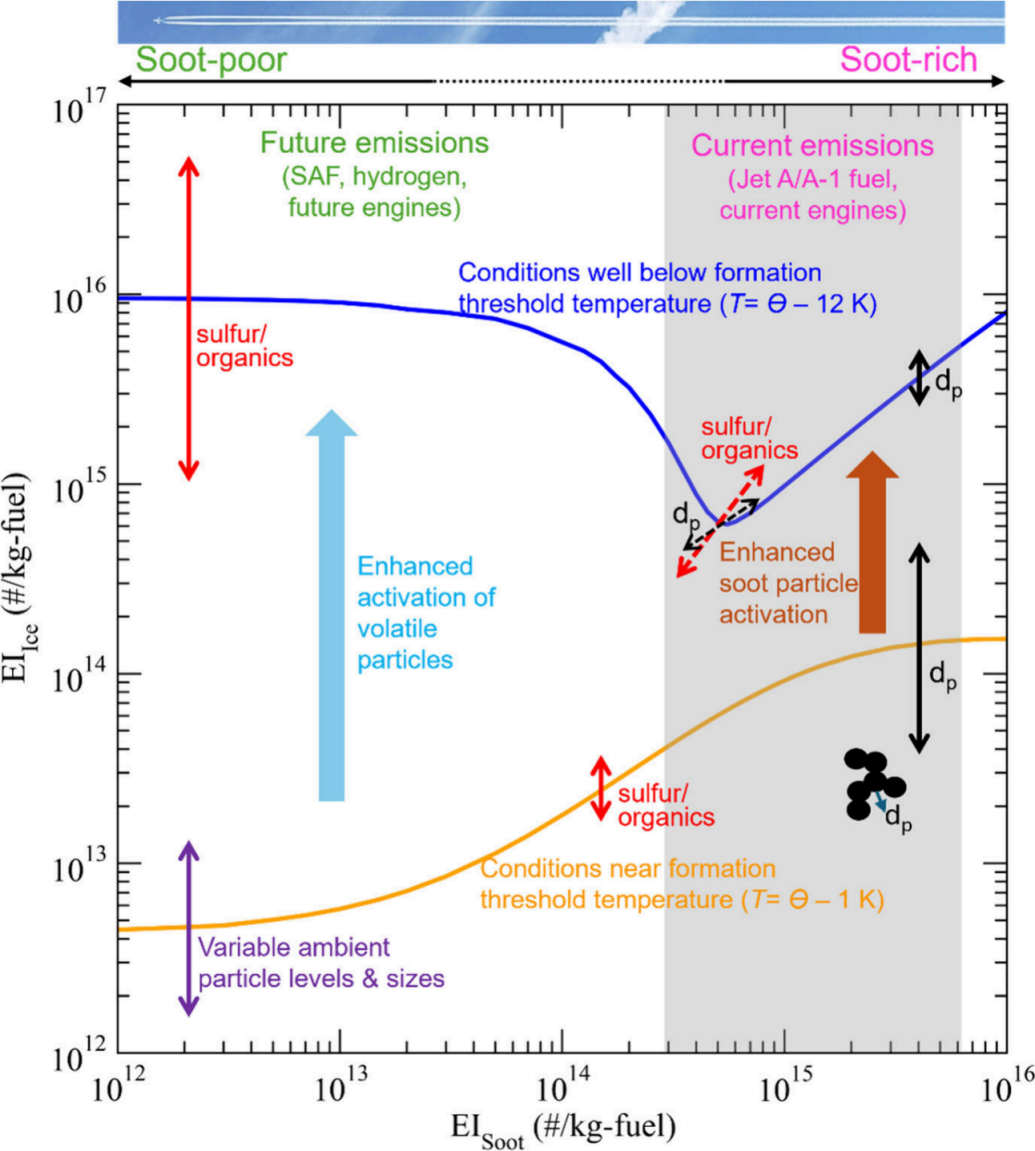
Schematic illustration showing the dependence of contrail ice crystal number emission index (EI_ice_, per kilogram of fuel burned) on the multiple dimensions of soot number emission index (EI_soot_), ambient temperature (*T*), diameters of primary soot particles (*d*_p_), fuel sulfur and concentrations of condensable organics (sulfur/organics), and number concentrations and sizes of ambient particles (see text for details).

Apparently a variety of factors affect the number concentrations of ice crystals formed in contrails. Figure 6 illustrates schematically the impacts of various factors on the contrail ice crystal number emission index (EI_ice_), derived from the simulations using the updated ACM model described in this paper. Two results are shown for an ambient temperature *T* close to (1 K) a contrail formation threshold temperature (orange curve), Θ ≈ 226 K, frequently found in extratropical cruise conditions, and a rather low temperature 12 K below this value (blue curve). At intermediate ambient temperatures, nucleated ice crystal numbers increase due to enhanced water activation of either soot or ultrafine volatile particles in the plume. For the two ambient temperature curves given in Figure 6, an FSC of 500 ppm, which is the average FSC of current jet fuels, is assumed. As pointed out earlier, condensable organics, while not yet considered in the present model simulations, may play a role similar to that of sulfur by contributing to the formation and growth of volatile particles. Sulfur/organics have a much stronger impact in soot-poor regimes when *T* is well below Θ (indicated by a longer red double arrow) and have a small but visible effect on soot activation in regimes with medium and high levels of soot particles when *T* is close to Θ (indicated by a shorter red double arrow). The dashed arrows show the impacts of sulfur/organics and *d*_p_ on the position of the minimum EI_ice_. Both the number concentrations and sizes of ambient particles have strong effects on EI_ice_ in the soot-poor regime when *T* is close to Θ (indicated by a purple double arrow).

It is noteworthy that *d*_p_ has a much stronger impact in soot-rich regimes when *T* is close to Θ (indicated by a longer black double arrow) than when *T* is well below Θ. In addition, when *T* is close to Θ (orange curve), the dependence of EI_ice_ on EI_soot_ in the soot-rich regime is nonlinear, and EI_ice_ begins to level off as EI_soot_ increases beyond ∼10^15^/kg-fuel. This nonlinear dependence, caused by the self-limiting effect of soot activation on RH_max_, was not indicated in our previous study.^2,9^ Further research is needed to evaluate these predicted features with in situ contrail measurements taken at ambient *T* close to Θ.

The results shown in Figures 5 and 6 indicate that the simultaneous reduction of EI_soot_ and *d*_p_ for 100% SAF or lean burning engines may lead to EI_ice_ as high as or higher than those of current flights using jet fuel. The removal of sulfur in the fuel is expected to reduce the sizes of volatile particles effectively and thus EI_ice_ in such situations, unless condensable organic species substantially contribute to the formation and growth of volatile particles. We emphasize that measurements of EI_ice_ in soot-poor conditions at various ambient conditions and FSC are needed to further constrain model predictions and assess the potential importance of organics species.

While the upgraded model confirms the overall (expected) dependence of EI_ice_ upon EI_soot_ in soot-rich conditions,^22^ our study closes in on a finer detail, namely the activated fraction of all emitted soot particles, which is made possible by more recent in-flight measurements. We confirm with detailed numerical simulations that partial water activation of emitted soot particles matters for contrail formation, indirectly confirming the homogeneous freezing scenario underlying our simulations and the “water saturation constraint”.^2^

One key uncertainty of this study with regard to the contributions of volatile particles to contrail ice formation is the possible role of condensable organics in the formation and growth of volatile particles, which is not considered due to the lack of relevant measurements during ECLIF1–3. The contribution of condensable organics to volatile particles and contrail ice particles is likely to be important when FSC is low and *d*_p_ is small, such as in the case of 100% SAF.^30^ As the aviation sector is moving toward decarbonization and mitigating the non-CO_2_ climate effects, it is critical to characterize the concentrations and properties of condensable organic species in the exhaust plume from modern engines running on SAF and understand their role in determining the properties of volatile particles and contrails formed under various conditions. We plan to expand the ACM model described in Section 2.2 to study the possible influence of condensable organics on volatile particles and associated impacts on contrail ice particle formation in the near future.

The effective radiative forcing of aviation is currently thought to be dominated by contrail cirrus.^4^ As the aviation industry is moving toward SAF and/or lean-burning engine technology which reduces both the emission index of nonvolatile soot particles and the primary soot particle sizes, it is important to understand how the combined changes may affect contrail formation and its climate impacts. Our new finding that contrail ice numbers may exceed EI_soot_ even in soot-rich and intermediate soot emission regimes has repercussions for global climate model studies and contrail mitigation efforts that relate to the use of lean combustion engines and alternative aviation fuels by adding a further source of uncertainty in simulations of contrail cirrus radiative forcing. Models that parametrize contrail ice formation from emitted soot particles based on their effective aggregate sizes and neglect the contribution of ultrafine volatile plume particles to the contrail ice number budget need to be updated. A possible roadmap to address these uncertainties includes (1) reliable measurements of EIs and size distributions of soot and volatile particles under cruise conditions and their impacts on contrails across various engine types and fuel compositions; (2) evaluation and, if necessary, improvement of model performance with in situ measurements, especially in the soot-poor regime as well as in the soot-rich regime with ambient temperature close to the contrail formation threshold; (3) physically based parametrizations of the dependence of contrail ice properties on soot emissions and volatile particle formation for various engine types, fuel compositions, and ambient conditions; and (4) global modeling with physics-based and validated parametrizations of sub-grid processes.

## Supporting Information Available

Measured variations of average soot primary particle diameter (*d*_p_) versus mean aggregate diameter (*D*_p_) for various aircraft engines under various operation settings, and the ratio of *d*_p_ to *D*_p_ (PDF)
