# Supplementary material for: Revisiting Contrail Ice Formation: Impact of Primary Soot Particle Sizes and Contribution of Volatile Particles
Source: Environ Sci Technol. 2024 Sep 26;58(40):17650–60. doi: 10.1021/acs.est.4c04340 (PMC11465739; doi:10.1021/acs.est.4c04340)
Supplement: Supplementary file 1 — es4c04340_si_001.pdf [file es4c04340_si_001.pdf]

Supporting Information (SI)

Revisiting contrail ice formation: Impact of primary soot particle sizes and contribution of volatile particles

Fangqun Yu[1], Bernd Kärcher[2], and Bruce E. Anderson[3]*

[1]Atmospheric Sciences Research Center, University at Albany, NY 12226, USA

[2] Institut für Physik der Atmosphäre, Deutsches Zentrum für Luft- und Raumfahrt, Oberpfaffenhofen, 82234 Wessling, Germany

[3] Science Directorate, NASA Langley Research Center, Hampton, VA 23666, USA

Number of pages: 3

Number of figures: 1

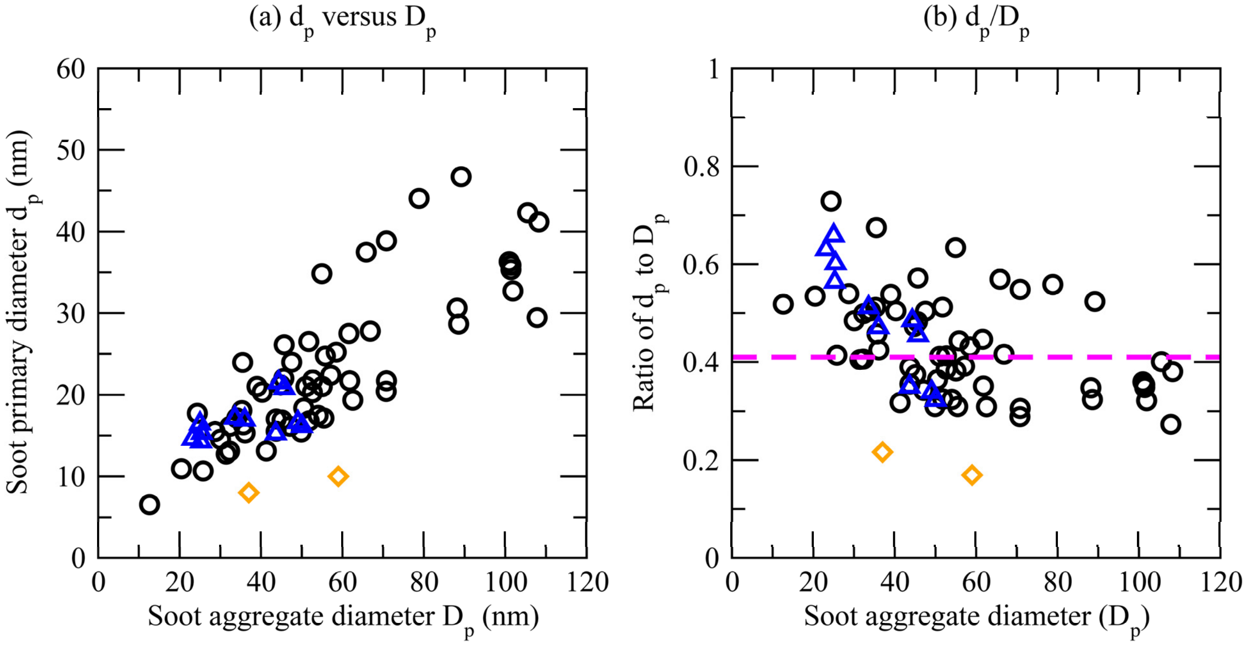

Figure S1. (a) Variations of average soot primary particle diameter (d_p) versus mean aggregate diameter (D_p) for various aircraft engines under various operation settings, as measured by Kumal et al. (2020) (black circles), Saffaripour et al. (2020) (blue triangles), and Kelesidis et al. (2023) (orange diamonds). (b) The ratio of d_p to D_p versus mean aggregate diameter (D_p) based on the data in (a). The pink dashed line shows the median d_p/D_p value.

REFERENCES

Kelesidis, G. AA, Nagarkar AA, Trivanovic UA, Pratsinis S. EA: Toward elimination of soot emissions from jet fuel combustion. Environ Sci Technol., 57, 10276–83, 2023.

Kumal, R. R., Liu, J., Gharpure, A., Vander Wal, R. L., Kinsey, J. S., Giannelli, B., Stevens, J., Leggett, C., Howard, R., Forde, M., Zelenyuk, A., Suski, K., Payne, G., Manin, J., Bachalo, W., Frazee, R., Onasch, T. B., Freedman, A., Kittelson, D. B., and Swanson, J. J.: Impact of Biofuel Blends on Black

Carbon Emissions from a Gas Turbine Engine, Energy Fuels, 34, 4958–4966, https://doi.org/10.1021/acs.energyfuels.0c00094, 2020.

Saffaripour, M., Thomson, K. A., Smallwood, G. J., and Lobo, P.: A review on the morphological properties of non-volatile particulate matter emissions from aircraft turbine engines, J. Aerosol Sci., 139, 105467, https://doi.org/10.1016/j.jaerosci.2019.105467, 2020.
